# Raman Spectroscopy: In Vivo Application for Bone Evaluation in Oral Reconstructive (Regenerative) Surgery

**DOI:** 10.3390/diagnostics12030723

**Published:** 2022-03-16

**Authors:** Eduard Gheorghe Gatin, Pal Nagy, Stefan-Marian Iordache, Ana-Maria Iordache, Catalin Romeo Luculescu

**Affiliations:** 1 Faculty of Medicine, University of Medicine and Pharmacy “Carol Davila”, 050474 Bucharest, Romania; 2 Faculty of Physics, University of Bucharest, 077125 Magurele, Romania; 3 Faculty of Dentistry, Semmelweis University, 1085 Budapest, Hungary; kardpali@gmail.com; 4 Optospintronics Department, National Institute for Research and Development for Optoelectronics—INOE 2000, 077125 Magurele, Romania; 5 National Institute for Laser, Plasma and Radiation Physics, CETAL, 077125 Magurele, Romania; catalin.luculescu@inflpr.ro

**Keywords:** Raman spectroscopy, periodontal disease, calcium phosphates, bone tissue analysis

## Abstract

The aim of this study was to evaluate the quality of the bone, revealing the different phases for calcified tissues independent of the medical history of the patient in relation to periodontitis by means of in vivo Raman spectroscopy. Raman spectroscopy measurements were performed in vivo during surgery and then ex vivo for the harvested bone samples for the whole group of patients (ten patients). The specific peaks for the Raman spectrum were traced for reference compounds (e.g., calcium phosphates) and bone samples. The variation in the intensity of the spectrum in relation to the specific bone constituents’ concentrations reflects the bone quality and can be strongly related with patient medical status (before dental surgery and after a healing period). Moreover, bone sample fluorescence is related to collagen content, enabling a complete evaluation of bone quality including a “quasi-quantification” of the healing process similar to the bone augmentation procedure. A complete evaluation of the processed spectra offers quantitative/qualitative information on the condition of the bone tissue. We conclude that Raman spectroscopy can be considered a viable investigation method for an in vivo and quick bone quality assessment during oral and periodontal surgery.

## 1. Introduction

There is high interest in periodontal disease because it represents a significant healthcare challenge. The genesis of human research endeavors in periodontology has sought a final goal of completely rebuilding lost tissues to be as close as possible to the original structure and functionality that were lost due to disease progression [1,2,3]. In order to achieve this objective, a bone tissue evaluation and investigation are mandatory.

Recently, Raman spectroscopy has found widespread use in biological and medical applications. Advantages for Raman spectroscopy are as follows: label-free, non-destructive and non-invasive method that provides information about the molecular composition and structure of a sample. The instrumentation and evaluation procedures have evolved, enabling a slow transition from demonstrations to in vivo examinations. This transition is connected with technological developments and tightly bound requirements for a successful implementation in a clinical environment, which is often difficult and requires good cooperation between physician (medical doctor, surgeon) and physicist/biophysicist. Today, Raman spectroscopy is rapidly emerging as a promising tool for biomedical analytics and clinical diagnostics, such as the detection and staging of cancer [4], and it has been validated in countless ex vivo studies [1,2,3,4,5,6,7,8,9,10,11,12].

As promising results were obtained for ex vivo (in vitro) applications [3], there has been a sustained effort to move Raman spectroscopy to clinical in vivo applications, where the method can unfold its full diagnostic potential [9].

New generation of materials offer a strong Raman background for optical fiber connections, but the development of those probes is highly complex and makes it harder to produce single use probes. A possible alternative is that probes have to be designed (adapted or using a tailored head) in a way that allows sustainable sterilization procedures, making the technology appropriate for introduction in a medical environment. Since fiber optic-based Raman probes are usually single-point sensors, important efforts have to be made to record only the spectrum of a needlepoint spot in the investigated body part (this implies a precise location for the optical biopsy) and to use more points to collect data for an average value [3,9,10].

Nowadays, the latest in vivo studies and applications of Raman spectroscopy, as an efficient tool for intraoperative assistance and for medical diagnostics on a variety of diseases and tissue types, are focused on general medicine as follows: cardiovascular and inflammatory disease and lung, breast, digestive and urinary tract, brain and skin cancer [9].

Regarding dentistry and oral surgery, most of the studies involving Raman spectroscopy reflect an interest in the investigation of dental enamel, periodontal ligaments or periodontal markers from saliva [1,10,11,12]. For those studies, investigation of samples was performed by using in vitro (ex vivo) protocols.

A prediction regarding a possible in vivo application of Raman technique in oral surgery was advanced recently [3]. The present study is a preliminary clinical investigation in order to identify bottlenecks and summarize future developments necessary to bring the emerging technology of Raman spectroscopy to clinical end users.

## 2. Materials and Methods

The present study selected a group of ten (10) patients who were under medical surveillance and had a very clear clinical status reported either as healthy, periodontal or with a history of previous periodontal conditions. The bone pieces harvested were part of routine clinical care, and details regarding the group of patients are listed in the table below (Table 1). According to medical assessments of the patients and status achieved, a color code was assigned to each one as follows: green (• periodontally healthy), blue (• history of periodontitis) and red (• periodontitis).

For every patient, a surgical procedure was indicated on an edentulous alveolar ridge site. At a start of our study, the Raman spectroscopy technique was used primarily on patients’ oral sites of interest for in vivo (viv) evaluation, and then, the same investigation was performed for the harvested bone samples from the same sites for the in vitro (vit) investigation.

### 2.1. In Vivo Measurements

In order to perform the in vivo investigation of the patients, an “adaptive cap” for the Raman probe was used, which is sustainable for steam autoclave sterilization according to standard protocol. This adaptive cap contains a slot for inserting the Raman probe equipped with a spacer sleeve that fits and holds the Raman probe in a fixed position.

Prior to examination, the selected area of the jawbone was prepared with blood suction, washed with saline and kept dry as much as possible. During data acquisition, there were no electromagnetic wave sources (no light) in order to avoid fluorescence contamination. The Raman probe was directed almost in a perpendicular position on the jawbone surface (interest site) chosen for examination. For each patient, two Raman spectra were recorded in vivo. We addressed the safety for the patient and operator by remotely switching ON the device only when the Raman probe was on the tissue surface (laser beam was not seen) and automatically OFF after measurement elapsed time. Additionally, the equipment comes with protective eyeglasses, but they were mostly used in the laboratory for in vitro experiments when we had to focus the Raman probe on the sample.

### 2.2. In Vitro Measurements

After in the vivo examination, bone cores were harvested with the help of a trephine (2 mm in diameter, Meisinger, Neuss, Germany). Due to the short time of preservation, all samples (1 mm^2^) were rinsed with standard saline and then stored in 70% ethylic alcohol solution after surgery. Prior the ex vivo investigation, samples were rinsed with pure water and then air dried [3].

All patients signed informed consent. The biopsy and Raman investigation protocol were approved by the Semmelweis University Regional and Institutional Committee of Science and Research Ethics (SE TUKEB No. 2020/141).

### 2.3. Characterization Methods

Raman spectroscopy evaluation was performed with a BTR111—785 RAMAN spectrometer device (λ = 785 nm, output power *p* = 300 mW and spectral resolution as fine as 4 cm^−1^) in the Raman shift range 300–1800 cm^−1^ for both in vivo and ex vivo bone samples investigation. The integration time was 1000 ms, and laser power was fitted for 10% from maximum output (300 mW). Raman spectrometer was calibrated with a Si (100) standard before and after data recording in the case of both in vivo and ex vivo measurements. Experimental data were recorded under the same geometrical conditions during bone sample evaluation, in two points and three points corresponding to in vivo and ex vivo measurements, respectively, in order to avoid local heating and florescence contamination from additional light sources. Data processing was performed by using OriginPro v2017 software (OriginLab, Northampton, MA, USA). Selected values for Raman peaks intensities were obtained after baseline correction, and unit normalization was applied to raw data (dark subtracted, not affected by noise, collected peak to peak). Differences in peaks intensity on raw spectra reflected the differences in the quantities of the chemical components for investigated specimens. Sensitive qualitative/quantitative information may be obtained according to the Raman spectra shape (including the fluorescence information) using raw data (no flat line subtraction, without smoothing) [3,10].

EDX (energy dispersive X-ray spectroscopy) and scanning electron microscopy (SEM) were also used for the characterization of harvested bones. The equipment employed in our study was a SEM microscope FEI Inspect S, equipped with a secondary electron detector in low vacuum and a solid state BSE detector, plus an auxiliary micro analytic SDD radiation detector. In order to avoid surface charge effects, after EDX investigation, the bone samples were coated with a 10 nm gold layer, and electron images were acquired [13,14]. Finally, principal component analysis (PCA) was performed on pre-treated data of the full spectral range, using the Minitab 18.1 software program in order to identify distinctions and outliners in the pattern.

## 3. Results

Raman investigation highlights the peaks (Raman shift) for the main bone (cortical or cancellous type) components (chemical groups and elements) in order to evaluate differences between bone tissue for the investigated patients (healthy, with a history of periodontitis or currently being diagnosed with periodontitis). The following list (Table 2), in order of increasing wavenumbers, shows the Raman bands (shifts) of bone tissue established to be relevant to our study and for future tracking and evaluation of the patients [3].

**Figure 1:**
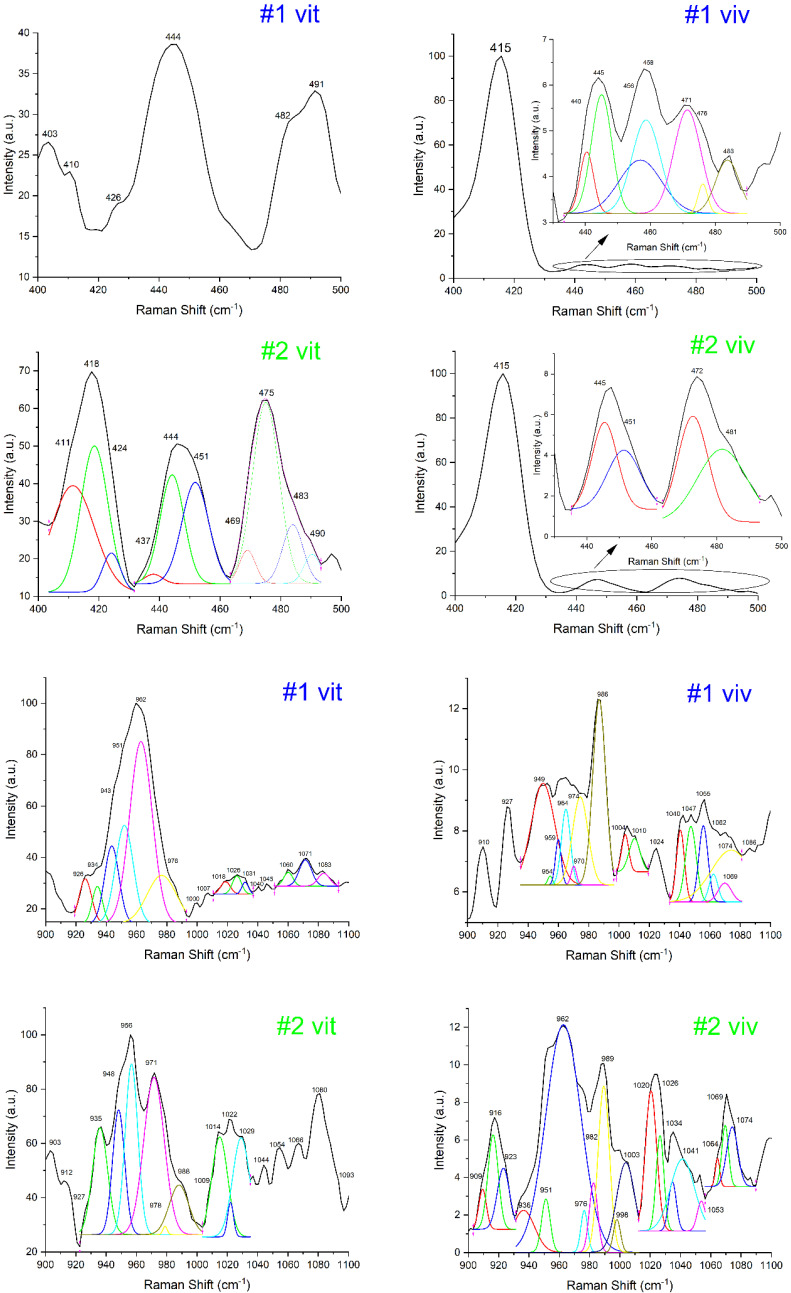
Raman spectra shown for patients 1 and 2. The spectra were accumulated both in vitro and in vivo, analyzed in two windows: 400–500 cm^−1^ and 900–1100 cm^−1^, presented as processed data and after deconvolution (new peaks, with different colors). The rest of the Raman spectra (patients 3–10) are presented in the Supplementary File. The numbering stands for: # patient no. in vivo/in vitro.

Regarding Raman investigation for the patients, both in vitro and in vivo, according to spectra from Figure 1 and Figure S1, the obtained results are summarized and depicted in Table 3.

The highest peaks for the Raman shift were obtained for the interval (950–970 cm^−1^), corresponding to sub-intervals (955–960 cm^−1^, HAP amorphous phase) and (960–965 cm^−1^, HAP crystalline phase). The rest of Raman bands have lower intensity values. This is one of the main characteristics obtained from both in vitro and in vivo experiments. Another characteristic is that all peaks intensities have lower values for in vivo measurements than in vitro measurements. This is presumably caused by blood and other residual tissues when operating in vivo and also by the fact that the measurements were not conducted perpendicular on the sample surface.

For Raman spectra evaluation and results discussion, the values we selected corresponded to the shift interval (955–965 cm^−1^) and (1020–1030 cm^−1^). We propose that values for HAP phases and PPi are the ones defining the bone quality evaluation (according to Table 2). Values from the other Raman shift intervals (Table 3) support our premise.

According to the patient status established in Table 1 and after a careful evaluation of Raman investigation results from Table 3, we noticed some “rules” regarding the rates (fractions) of pyrophosphate peak intensities reported to HAP phases peak intensities. The Raman results presentation and discussion were systematized according to patient medical status after the clinical evaluation. We have the following categories:

(I) Clinical status—periodontally healthy (patients: #2, #6 and  #9)

Regarding this category, we observed two different situations:

(i) Both HAP phases (amorphous—corresponding to immature bone and crystalline—corresponding to mature bone) associated with Raman shift intervals (955–965 cm^−1^) and specific values as indicated in Table 2 were detected. Generally peak intensity corresponding to immature bone is higher than that corresponding to mature bone, as for patients’ #6vit and #9viv. As such, we obtained similar values for patient #6viv. The difference is made by the ratio of pyrophosphate intensity reported [3] to that corresponding to amorphous HAP phase (immature bone). The ratio belongs in the interval (0.45–0.80).
(ii) Only one HAP phase is clearly distinguished. The ratio of PPi (pyrophosphate) intensity reported to that corresponding to HAP phase (amorphous/crystalline) belongs in the interval (0.40–0.80) for patient (#2vit/viv) or no pyrophosphate (small quantity) detected for HAP crystalline for patient (#9vit).

(II) Clinical status—Previous periodontitis (patients: #1, #3, #4, #5 and #8)

Because those patients are considered cured, this category shows intensities similar to the healthy one. Some small differences are observed and can be categorized into two different situations:

(i) Both HAP phases (amorphous—corresponding to immature bone and crystalline—corresponding to mature bone) associated with Raman shift intervals (955–965 cm^−1^) and specific values as indicated in Table 2 were detected. Peak intensity corresponding to the immature bone is higher than that for mature bone, and the ratio of PPi intensity belonging to immature bone belongs in the interval (0.40–0.70), as for patients’ #1viv, #4viv and #5vit.
(ii) Only one HAP phase is detected, corresponding to either immature or mature bone. The ratio for PPi is lower than for (i) category and belonging to the interval (0.20–0.60), when just immature phase is detected, as observed for patients’ #3vit, #4vit and #8vit. The value for the PPi ratio can even be in the thousandths scale for a very small peak of immature bone, as for patient #3viv.

When just the mature phase is detected, the peak intensity corresponding to PPi is much lower and belonging in the interval (0–0.40), as was noticed for patients’ #1vit, #5viv and #8viv. We do not consider relevant the ratio of PPi reported to mature phase, and we are focused on the ratio reported for immature bone phase, according to [3].

(III) Clinical status—Periodontitis (patients: #7 and #10)

Concerning this category of patients, we also observed two situations, but this time the imbalance between components ratio was significantly different. The two situations are as follows:

(i) Both HAP phases (corresponding to immature/mature bone) are defined and belonging in the Raman shift interval (955–965 cm^−1^), but the ratio of PPi belonging to immature bone (or mature bone) is supra-unitary. For example, for patient #7vit, the value obtained for this ratio was 1.41.
(ii) The HAP phases are not clearly defined, and the Raman shift is 959/960 cm^−1^. Under these conditions, the ratio of PPi compared with that of HAP phase belongs between (0.15–0.80), with a trend for lower or higher values, depending on the type of measurement conditions (0.18, 0.77, 0.70), as for patients (#10viv, #10vit, #7viv).

An important support for bone quality evaluation is offered by the Raman peaks belonging to the shift interval (1070–1080 cm^−1^) that is associated with intense B-type carbonate bands (1070 cm^−1^ assigned to CO_3_^2−^ (ν_1_) and 1076 cm^−1^ assigned PO_4_^3−^ (ν_3_) [15].

The two sites for carbonate substitution in apatite each control the crystal’s perfection and crystallite size, according to different environmental conditions. The B-type carbonate is specific for apatite formation of biominerals under physiological conditions [20,21].

We presume that peaks intensity assigned to B-type carbonate (shift interval 1070–1080 cm^−1^) can be associated with a “physiological parameter as an intensity” regarding bone tissue metabolism, keeping the balance between mature/immature bone phases.

The ratio of B-type carbonate bands intensity reported to HAP phase detected (corresponding to mature/immature bone) are as follows:

(I) Clinical status—Periodontally healthy (patients: #2, #6 and  #9)

Regarding this category, the ratio belongs to the interval (0.40–0.90). Those values correspond to a metabolic activity from medium to a high level.

(II) Clinical status—Previous periodontitis (patients: #1, #3, #4, #5 and #8)

Regarding these patients, the ratio belongs to the same interval as the healthy category. There were noticed two exceptions, as 1.11 for patient #3vit and 0.21 for patient #8vit. Those exceptions are similar to values obtained for periodontal patients, but these patients are periodontal “healed” (we assume that the healing may not be complete).

(III) Clinical status—Periodontitis (patients: #7 and #10)

For patients assigned to this category, the ratio belongs to the intervals (0.30–0.45 and 0.70–1.05) with no average values. The unbalance between HAP phases is confirmed.

An important remark regarding the results depicted in Table 3 and previous results is that the peak intensities for PP_i_ and B-type carbonate bands have the same order of magnitude and similar values. Thus, the level of PP_i_ is relevant and can be considered a marker of the process dynamics for phase transition HAP (amorphous phase, immature bone)→HAP (crystalline phase, mature bone) [3].

Regarding the results depicted in Table 3 as a general feature, all values respect the same tendency (behavior) for both in vivo as well as in vitro measurements.

For the calcium phosphates compounds involved in the phase transition process (immature bone→mature bone), the amount of P is constant (6 atoms), and the additional 2 Ca atoms “captured” during the phase changing process are making the difference between the amorphous/crystalline phase HAP. The ratio Ca/P is the parameter characterizing the phases: 1.33—corresponds to the amorphous phase; 1.66—corresponds to the crystalline phase HAP, while values between those extremes correspond to the phase balance mixture. The EDX results depicted in (Table 4) support the Raman results presented above [3].

For healthy patients (#2, #6, #9) and previous periodontal patients (#1, #3, #4, #5, #8), we noticed a balance between the amorphous/crystalline phase, with higher ratio values for those belonging to the healthy category and all values being strictly above-unit (>1). For periodontal patients (#7 and #10), the imbalance between Ca/P rates is evident, and the obtained values are strictly below-unit (<1).

**Figure 2:**
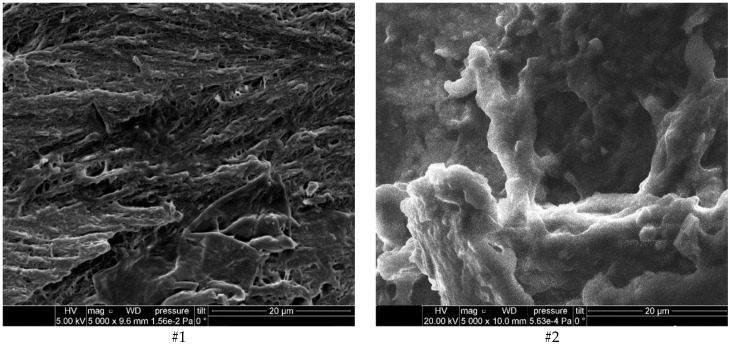

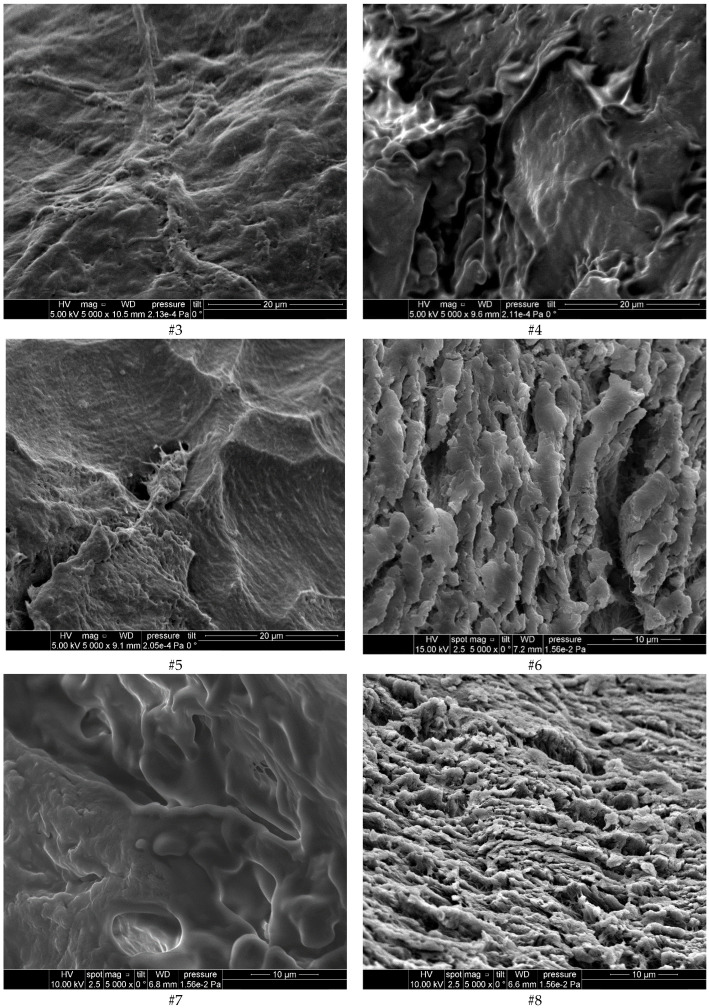

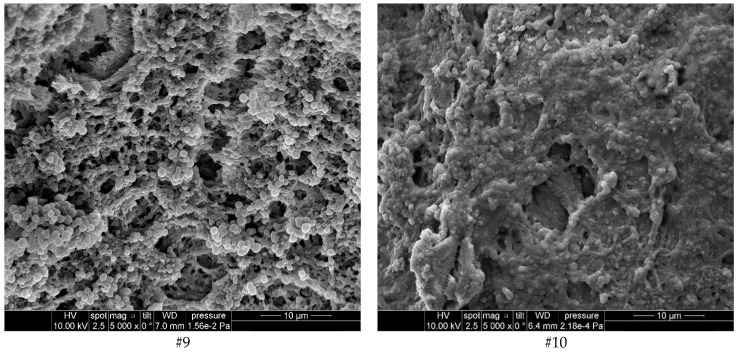
Morphological aspects of bone samples highlighted by SEM micro photos (patients’ #1–#10, magnification 5 × 10^3^).

Morphological aspects of bone samples highlighted by SEM micro photos (Figure 2, all obtained under the same conditions and magnification 5000×) are confirming the Raman results.

A lamellar and quite regular structure (tile shape with HAP crystalline formations) of bone tissue (mineral bone, MB) is noticed for patients #2, #6, and #9, belonging to the healthy category.

Non-regular and compact bone structure (immature bone, IMB) areas associated with small sectors of regular structure (mature bone, MB) are noticed for patients #1, #3, #4, #5 and #8 registered with previous periodontal problems and healed. Blurred and loosely organized crystalline phase sectors can be evidenced.

A compact bone structure, but with an unbalanced ratio between HAP phases (amorphous/crystalline) is observed for patients #7 and #10 belonging to the periodontal category. The SEM micro photos for those patients’ samples are “crystal clear” pictures of the Raman results discussion section. A larger sector corresponding to IMB (immature bone) than that of MB (mature bone) with a little regular structure is observed for patient #7, confirming the higher ratio (1.41) obtained for PP_i_. Regarding patient #10, the SEM micrograph evidenced some HAP formations but with HAP phases not clearly defined.

**Figure 3:**
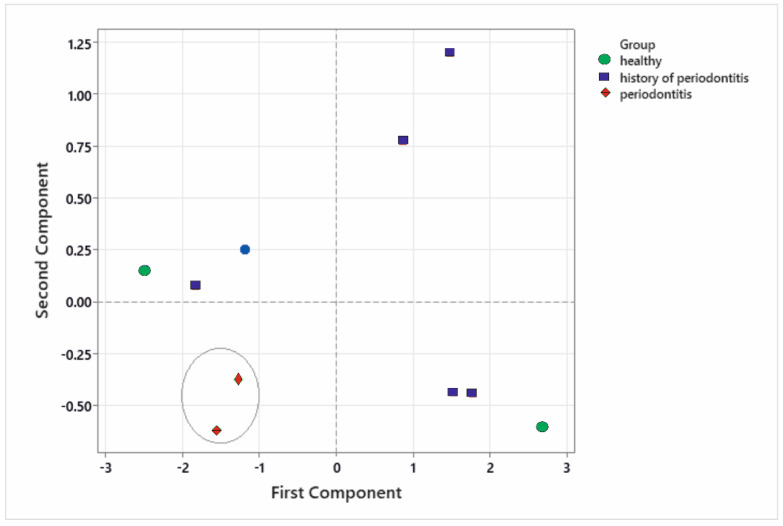
Principal components analysis performed on the Raman spectra showing the grouping of the patients in relation to the peak intensities.

Performing PCA (Figure 3) on the Raman data showed that the patients with periodontitis were clustered together, while the healthy group and the group with a history of periodontitis showed a mixed clustering. More precisely, patient #2 and patient #8 were attributed to different groups than the groups they actually were a part of (patient #2 who had a history of periodontitis was associated with the healthy group, and patient #8 who was healthy was associated with the other group). The reason behind the mixed clustering of the healthy and healed patients is the fact that their bone structure is very similar. This is important when assessing the status of the patient as healthy vs. periodontitis, particularly since the first four components of our analysis explain 98.2% of the variation in the data. However, further analysis is needed to fine-tune the resolution for a more detailed assessment: healthy/healed/diseased.

Strengths: We characterized both in vivo and ex vivo 10 samples of bone (healthy, healed and with periodontitis) using Raman spectroscopy. We identified important differences between HAP structure (crystalline/amorphous) corresponding to mature/immature bones and connected them to the health status of the patients. We successfully managed to differentiate between healthy patients and patients with periodontitis. The Raman results were compared with EDX and SEM, which managed to connect the structure shown in the Raman spectra with the topography and composition ratio shown in the EDX-SEM. 

Limitations: Considering the limitations of this study, we lack in-depth multivariate analysis. Although we performed PCA, further statistical analysis are needed because there is a rich amount of data that has not been fully researched (e.g., connecting the Raman data with the type of structure shown in SEM and the Ca/P fraction, as well as with other descriptive analysis, such as computed tomography [22,23,24]). Increasing the number of patients will help in determining the differences between healthy bones and healed bones.

## 4. Conclusions

The present study aimed to promote this new simple, quick, noninvasive and independent method of investigation based on Raman spectroscopy for in vivo application in oral reconstructive surgery regarding evaluation of healing in the bone. The results obtained by Raman spectroscopy (Table 3) are supported by EDX results (Table 4) and are confirmed by SEM micro photos.

A careful analysis of Raman spectra may offer quantitative results for bone samples—as forensics, taking into consideration different targeted Raman intervals/windows for different bone specimens (Table 2). A larger database containing more patients will help to establish better threshold values for the chemical compounds present in the sample. This method represents an improvement in the evaluation of the bone regeneration process in oral/reconstructive surgery over those based on X-rays or even histology.

Take-home messages: 1. Raman spectroscopy can discern between healthy patients and patients with periodontitis; 2. This technique can measure the degree of healing of the bone, but further research is needed to properly evaluate between healthy and healed bone tissue; 3. It is simple, fast and noninvasive, offering clear advantages compared to X-ray and histology.

## Figures and Tables

**Table 1:** List of patients involved in the study with clinical remarks; specifications regarding bone samples. Legend: (• periodontally healthy), (• history of periodontitis), (• periodontitis).

Patient Number	Gender	Age (Years)	Healthy	Periodontal Condition	Bone Phenotype	Bone Type
#1	M	58	-	History of periodontitis	thick	more cortical
#2	M	70	Yes	-	thick	more cortical
#3	M	64	-	History of periodontitis	thin	more cortical
#4	F	50	-	History of periodontitis	thick	more cortical
#5	M	70	-	History of periodontitis	thin	more cortical
#6	M	35	Yes	-	thin	more cortical
#7	F	62	-	Yes	thin	more cancellous
#8	F	37	-	History of periodontitis	thin	cortical/cancellous
#9	F	45	Yes, lower jaw	Upper jaw	thin	more cortical
#10	M	43	-	Yes	thick	more cortical

**Table 2:** Targeted Raman shift for bone specimens.

Raman Shift	Characteristics	Assignment	References
430–450 cm^−^^1^	very strong	ν_2_ PO_4_^3−^, shoulder	[15,16]
955–960 cm^−^^1^ 955 cm^−^^1^ 957 cm^−^^1^	very strong	Extensive mineral immature bone; ν_1_ PO_4_^3−^, P–O phase; ν_1_ PO_4_^3−^, extensive HPO_4_^2−^	[16]
960–965 cm^−^^1^ 963 cm^−^^1^	very strong	Mineral mature bone; ν_1_ PO_4_^3−^ tetrahedral internal mode	[16]
1023 cm^−^^1^	strong	PPi (P_2_O_7_^4−^), inorganic pyrophosphate; symmetric P••O stretch modes of PO_3_^2−^ moieties; ν_S_ PO_3_ and of P–O–P bridging	[17,18,19]
1070 cm^−^^1^ 1076 cm^−^^1^	strong	Mineral bone B-type carbonate HAP; CO_3_^2−^ (ν_1_) overlap; PO_4_^3−^ (ν_3_) overlap	[15]

**Table 3:** Peaks intensities according to Raman shift. Summarized results: normalized, average values (two/three points for in vivo */vitro *, data acquisition), standard deviation SD included.

Patient Number/Raman Shift (cm^−^^1^)/Normalized Intensity (%)/SD		430–450 cm^−^^1^	955–965 cm^−^^1^	1020–1030 cm^−^^1^	1070–1080 cm^−^^1^
#1	vit *	444→38.3%, SD = 2.01	962→99.3%, SD = 1.52	1026→35.3%, SD = 1.52	1071→40.2%, SD = 1.52
	viv *	445→6.2%, SD = 0.31	958→9.8%, SD = 0.51 964→8.9%, SD = 0.31	1024→7.4%, SD = 0.58	1074→7.4%, SD = 0.38
#2	vit	444→52.3%, SD = 2.08	956→88.4%, SD = 0.57	1022→68.8%, SD = 1.52	1080→78.1%, SD = 1.73
	viv	445→7.4%, SD = 0.32	962→12.07%, SD = 0.58	1023→9.4%, SD = 0.51	1074→6.9%, SD = 0.28
#3	vit	435→45.3%, SD = 1.52 443→53%, SD = 1	957→70.3%, SD = 1.52	1022→48.17%, SD = 1.15	1076→78%, SD = 1
	viv	438→3.9%, SD = 0.21	958→7.81%, SD = 0.33		1071→3.9%, SD = 0.27
#4	vit	445→31.3%, SD = 1.52	958→99%, SD = 1	1024→32.5%, SD = 0.57	1076→37.6%, SD = 0.57
	viv	440→5.4%, SD = 0.21	956→11%, SD = 1 962→7.51, SD = 0.33	1024→6.08%, SD = 0.31	1080→6.5%, SD = 0.27 1072→6.2%, SD = 0.21
#5	vit	442→46%, SD = 1	956→90.6%, SD = 1.15 965→62.8%, SD = 1.15	1027→55%, SD = 1	1075→58.6%, SD = 1.15 1079→69%, SD = 1
	viv	440→12%, SD = 1	961→23.7%, SD = 0.57	1024→12%, SD = 1	1072→22.5%, SD = 0.25
#6	vit	434→19.6%, SD = 1.15 440→15%, SD = 1	958→27.2%, SD = 0.25 964→26.2%, SD = 0.31	1023→15%, SD = 0.25	1073→20.5%, SD = 1.15
	viv	442→6%, SD = 1	955→28.5%, SD = 1.15 961→30.1%, SD = 1.51	1023→13%, SD = 1	1080→16%, SD = 1
#7	vit	433→16%, SD = 1	956→18.3%, SD = 0.27 964→14.3%, SD = 0.21	1023→25.9%, SD = 0.31	1073→19%, SD = 1
	viv	433→3.5%, SD = 0.22 438→6.5%, SD = 0.55	959→13%, SD = 1	1024→9.1%, SD = 0.34	1070→10.5%, SD = 0.25
#8	vit	435→7.2%, SD = 0.25 441→7.5%, SD = 0.38	957→24.4%, SD = 0.21	1023→15%, SD = 0.20	1071→5.2%, SD = 0.31
	viv	433→3.8%, SD = 035 438→6.5%, SD = 0.24	961→27%, SD = 1	1023→6.6%, SD = 0.50	1069→10%, SD = 1 1078→8.7%, SD = 0.38
#9	vit	435→6.5%, SD = 0.25 441→5.2%	963→13.5%, SD = 0.35		1070→6.2%, SD = 0.31
	viv	436→3.3%, SD = 0.31 441→4%, SD = 1 448→3.5%, SD = 0.33	957→13.5%, SD = 0.31 963→8.6%, SD = 0.20	1023→5.7%, SD = 0.50	1077→5.5%, SD = 0.25
#10	vit	443→13%, SD = 1 448→17.2%, SD = 0.25	959→34.2%, SD = 1.52	1022→26.4%, SD = 0.32	1071→15%, SD = 1
	viv	445→5.5%, SD = 0.20	959→30%, SD = 1	1023→5.6%, SD = 0.25	1079→9%, SD = 1

* means that for the first patient only two points were selected for the calculation of the average value.

**Table 4:** Ca/P fraction; summarized results according to the EDX method (four measurements each sample, standard deviation STD included).

Patient Number	Ca/P Ratio W/A	Mean Value	STD
#1	1.73, 2.01, 1.63, 1.85 1.34, 1.55, 1.26, 1.43	1.805 1.395	0.141 0.107
#2	1.49, 1.46, 1.39, 1.41 1.39, 1.38, 1.48, 1.41	1.437 1.415	0.039 0.039
#3	0.66, 1.06, 1.46, 0.92 0.52, 0.81, 1.13, 1.02	1.025 0.870	0.289 0.232
#4	1.46, 1.43, 1.75, 1.44 1.16, 1.21, 1.47, 1.31	1.520 1.287	0.133 0.118
#5	0.95, 1.45, 0.64, 1.03 0.77, 1.11, 1.30, 0.98	1.017 1.040	0.289 0.193
#6	2.39, 3.17, 2.30, 2.40 1.85, 2.47. 1.79, 1.96	2.565 2.017	0.351 0.268
#7	0.68, 0.99, 0.62, 1.24 0.52, 0.77, 0.47, 0.96	0.882 0.680	0.249 0.197
#8	3.04, 2.55, 2.97, 2.94 2.30, 1.96, 2.29, 2.27	2.875 2.205	0.191 0.141
#9	2.23, 0.92, 2.37, 2.48 1.72, 0.72, 1.82, 1.91	2.000 1.540	0.629 0.479
#10	0.53, 1.21, 1.09, 0.91 0.68, 0.95, 0.61, 0.58	0.935 0.705	0.296 0.168

## Supplementary Materials

The following supporting information can be downloaded at: https://www.mdpi.com/article/10.3390/diagnostics12030723/s1, Figure S1. Raman investigation for the patients 3–10, both in vitro and in vivo, analyzed in two windows: 400–500 cm^−1^ and 900–1100 cm^−1^. The numbering stands for: # patient no. in vivo/in vitro.
