# Supplementary material for: Raman Spectroscopy: In Vivo Application for Bone Evaluation in Oral Reconstructive (Regenerative) Surgery
Source: Diagnostics (Basel). 2022 Mar 16;12(3):723. doi: 10.3390/diagnostics12030723 (PMC8947687; doi:10.3390/diagnostics12030723)
Supplement: Supplementary file 1 [file diagnostics-12-00723-s001.zip › diagnostics-1590953-supplementary.pdf]

Raman Spectroscopy: In vivo Application for Bone Evaluation in Oral Reconstructive (Regenerative) Surgery

Eduard Gheorghe Gatin [1,2,*], Pal Nagy [3], Stefan-Marian Iordache [4,*], Ana-Maria Iordache [4,*] and Catalin Romeo Luculescu [5]

[1] Faculty of Medicine, UMF Carol Davila, Bucharest, Romania
[2] Faculty of Physics, University of Bucharest, Magurele, Romania
[3] Faculty of Dentistry, Semmelweis University, Budapest, Hungary; kardpali@gmail.com
[4] Optospintronics Department, National Institute for Research and Development for Optoelectronics—INOE 2000, Magurele, Romania
[5] National Institute for Laser, Plasma and Radiation Physics, CETAL, Magurele, Romania; catalin.luculescu@inflpr.ro
* Correspondence: masterdent2009@yahoo.com (E.G.G.); stefan.iordache@inoe.ro (S.-M.I.); ana.iordache@inoe.ro (A.-M.I.)

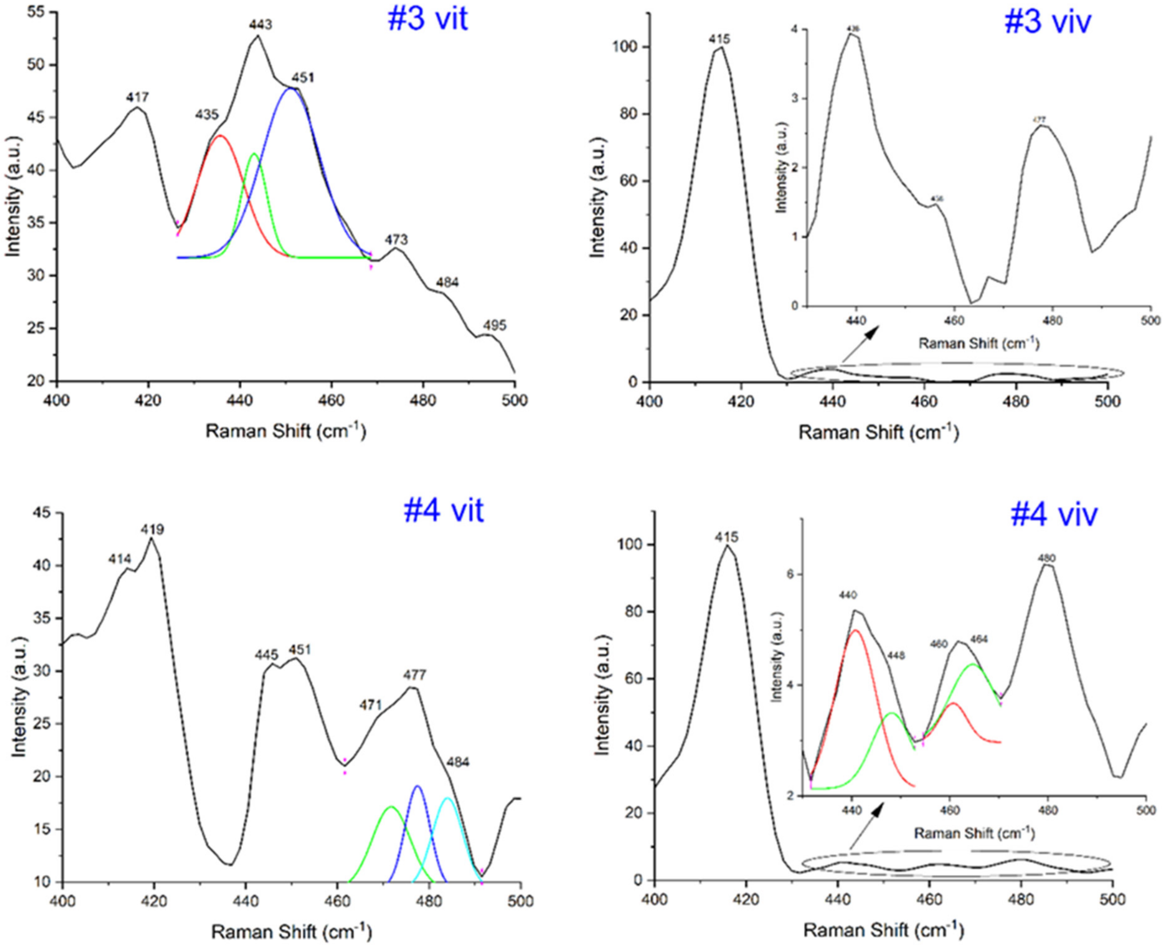
#3 vit
#3 viv
#4 vit
#4 viv
Intensity (a.u.)
Raman Shift (cm-1)
417
435
443
451
473
484
495
415
477
414
419
445
451
471
477
484
415
440
448
460
464
480

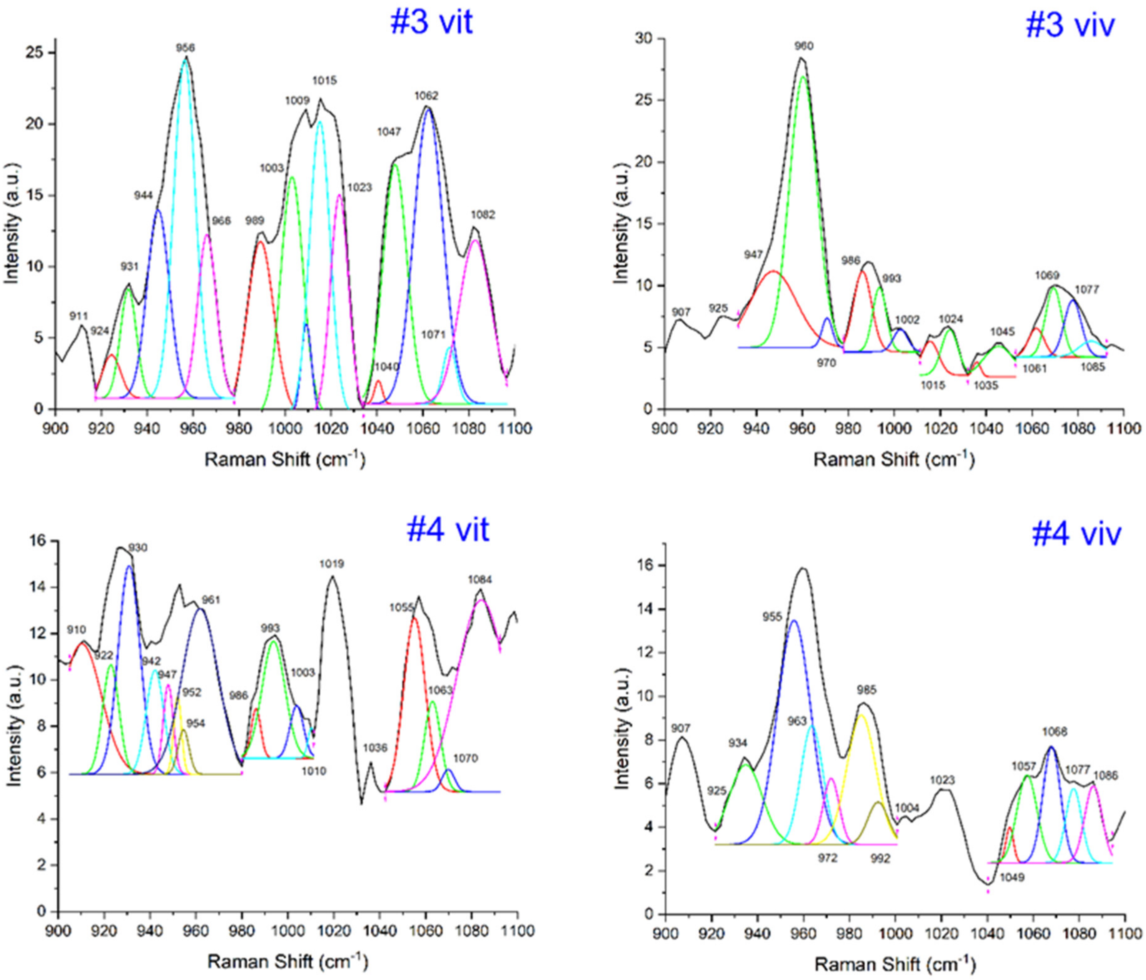
#3 vit
Intensity (a.u.)
Raman Shift (cm-1)
911
924
931
944
956
968
989
1003
1009
1015
1023
1040
1047
1062
1071
1082
#3 viv
Intensity (a.u.)
Raman Shift (cm-1)
907
925
947
960
970
986
993
1002
1015
1024
1035
1045
1061
1069
1077
1085
#4 vit
Intensity (a.u.)
Raman Shift (cm-1)
910
922
930
942
947
952
954
961
986
993
1003
1010
1019
1036
1055
1063
1070
1084
#4 viv
Intensity (a.u.)
Raman Shift (cm-1)
907
925
934
955
963
972
985
992
1004
1023
1049
1057
1068
1077
1086

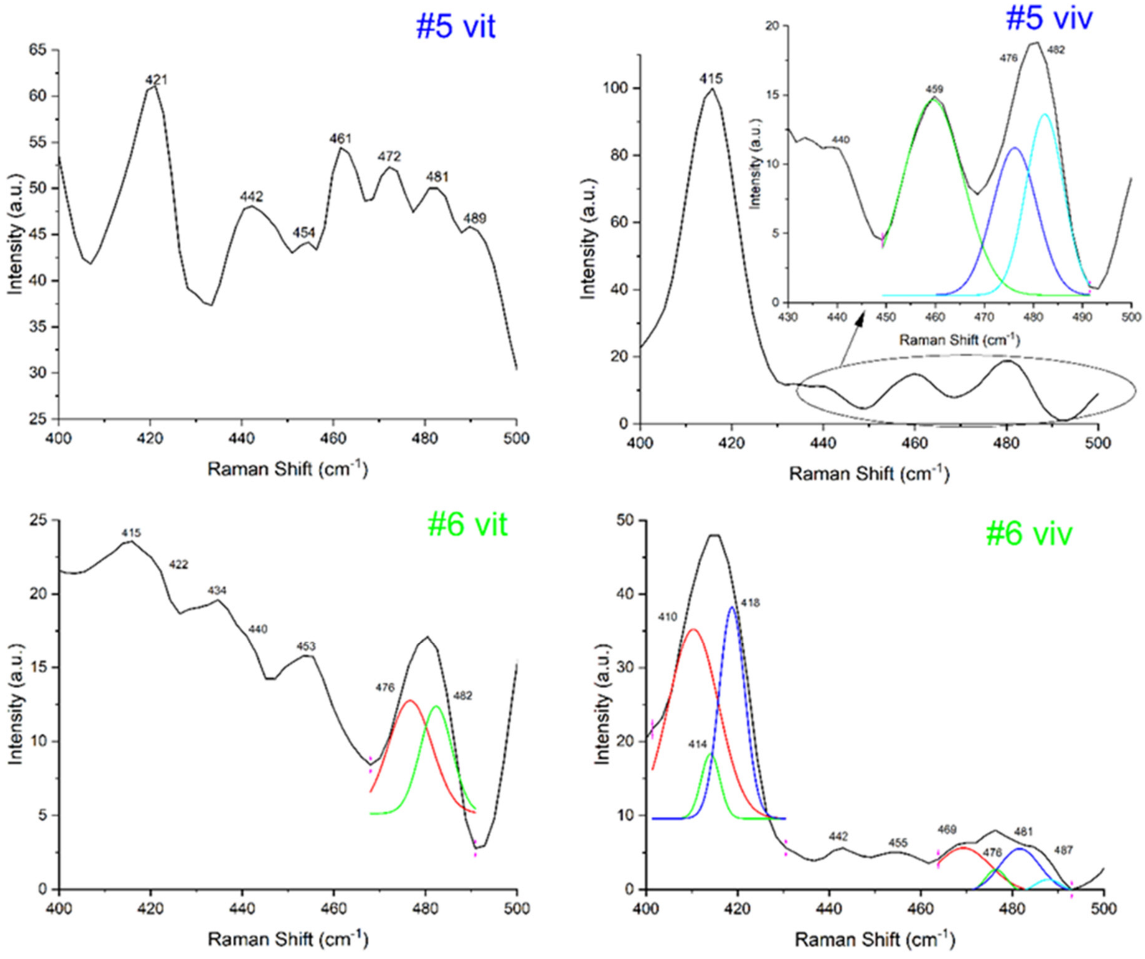

#5 vit
#5 viv
#6 vit
#6 viv
Intensity (a.u.)
Raman Shift (cm-1)

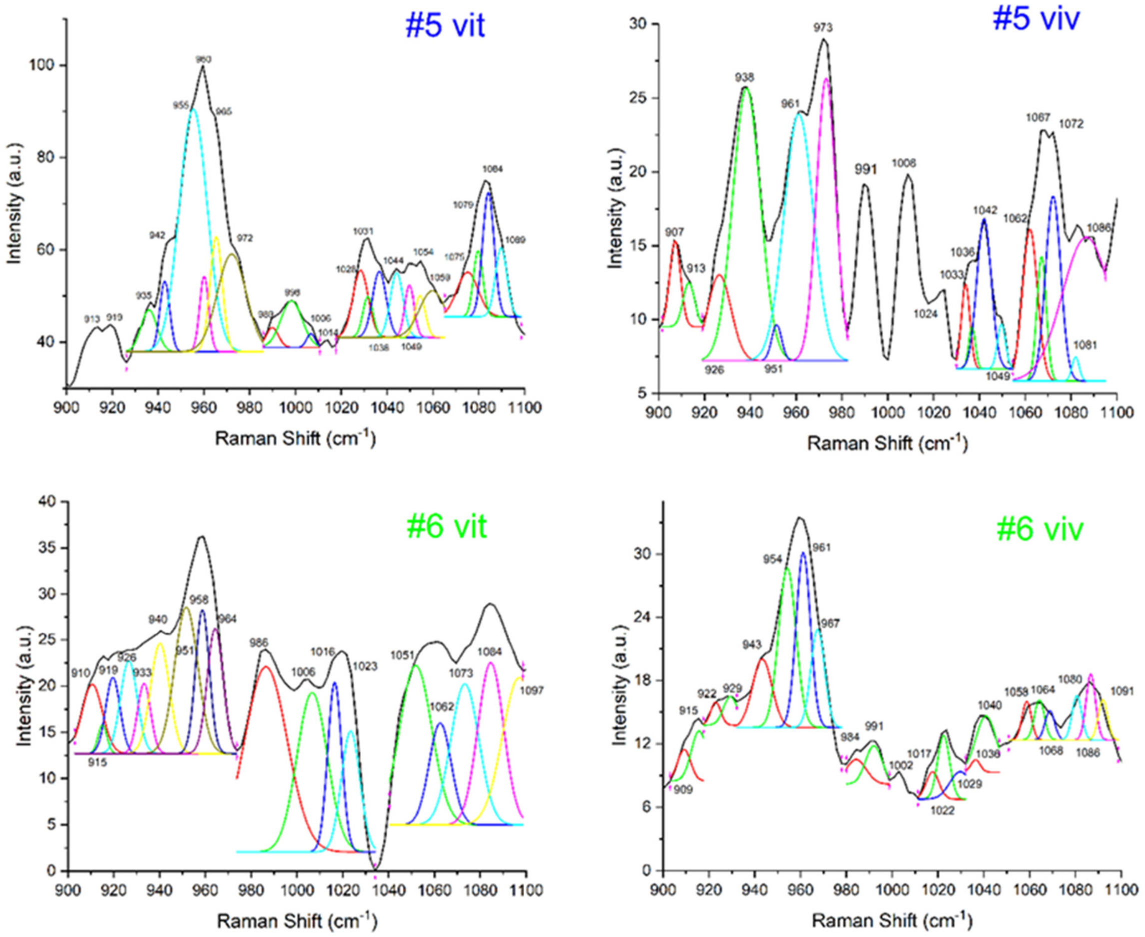
#5 vit
Intensity (a.u.)
Raman Shift (cm-1)
#5 viv
Intensity (a.u.)
Raman Shift (cm-1)
#6 vit
Intensity (a.u.)
Raman Shift (cm-1)
#6 viv
Intensity (a.u.)
Raman Shift (cm-1)

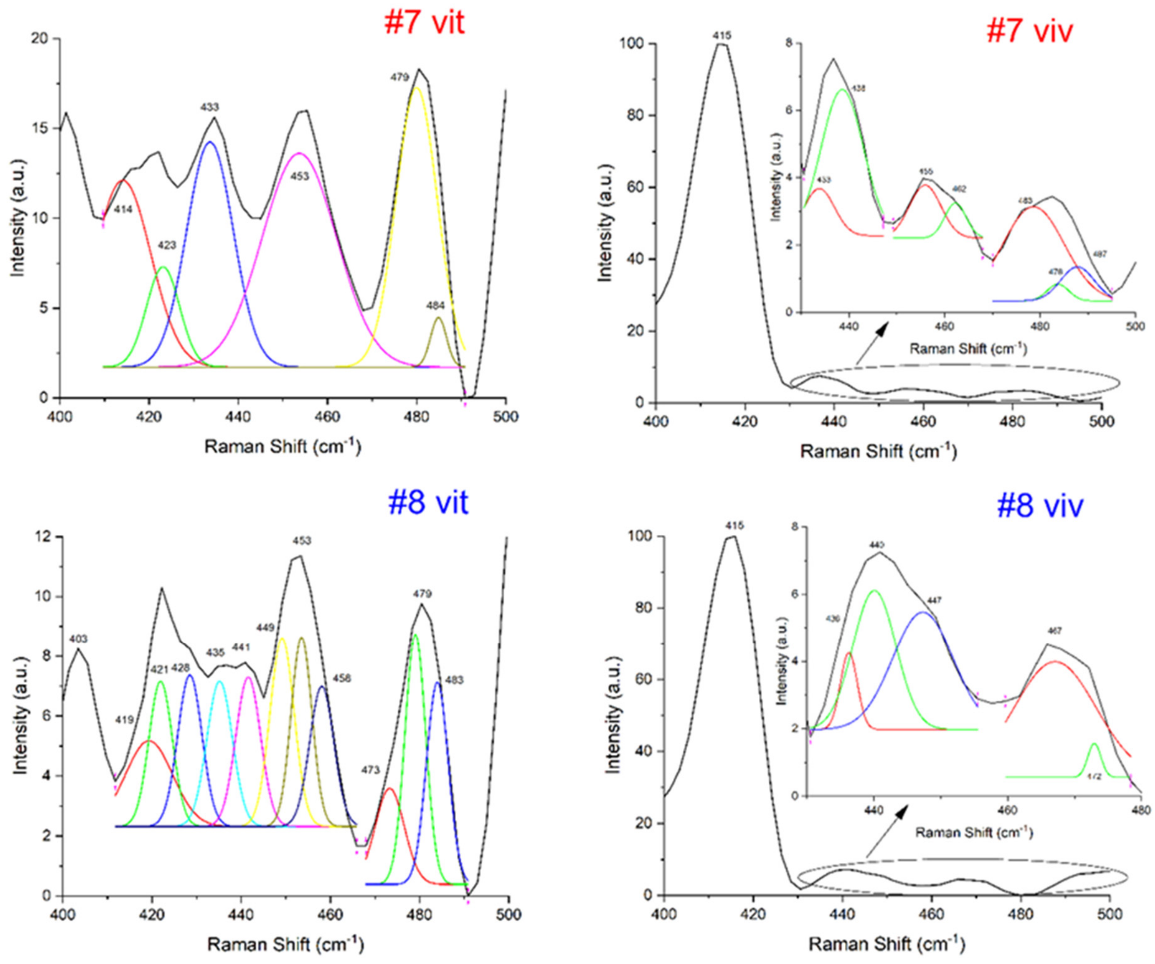
#7 vit
Intensity (a.u.)
Raman Shift (cm-1)
414
423
433
453
479
484
#7 viv
415
438
433
455
462
483
476
487
#8 vit
403
419
421
428
435
441
449
453
456
473
479
483
#8 viv
415
440
436
447
467
472

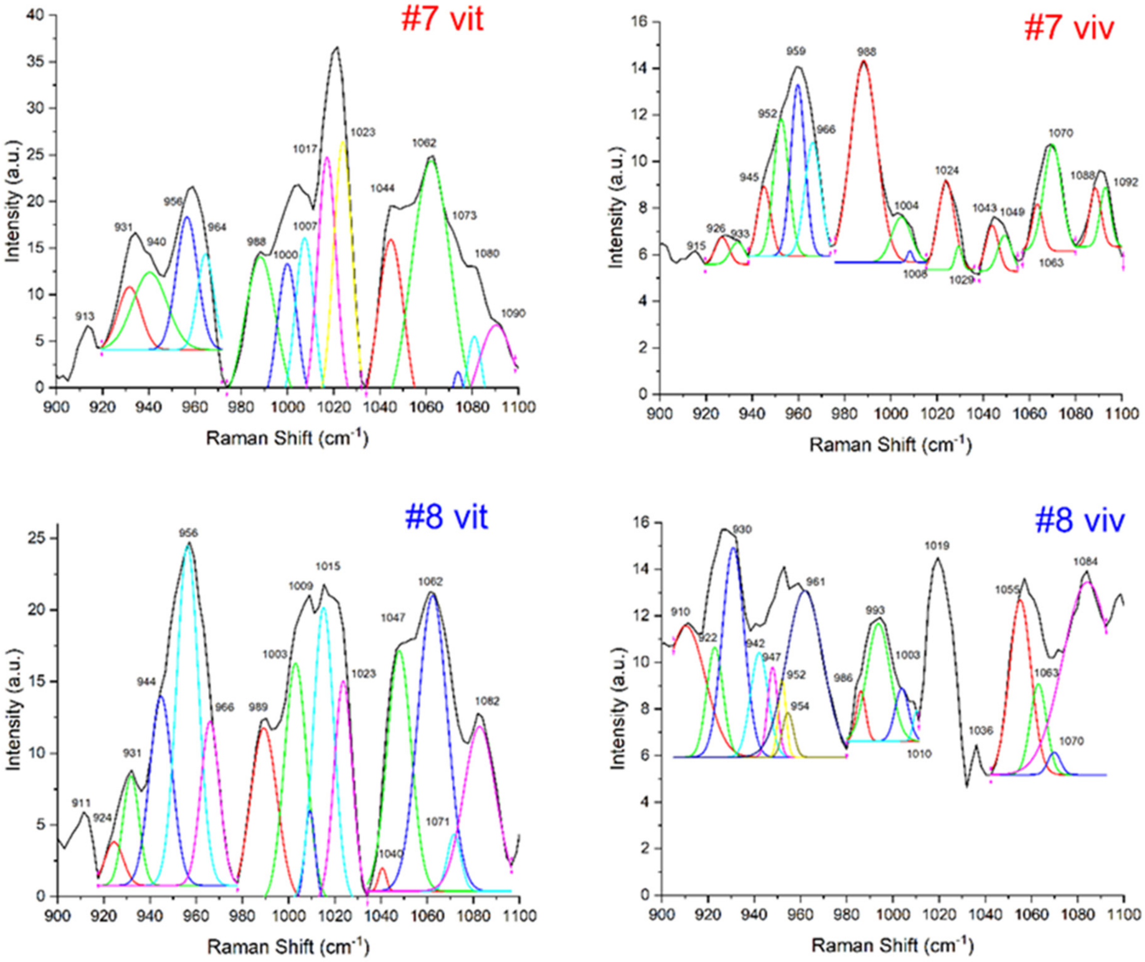
#7 vit
#7 viv
#8 vit
#8 viv
Intensity (a.u.)
Raman Shift (cm-1)
913
931
940
956
964
988
1000
1007
1017
1023
1044
1062
1073
1080
1090
915
926
933
945
952
959
966
988
1004
1008
1024
1029
1043
1049
1063
1070
1088
1092
911
924
944
966
989
1003
1009
1015
1040
1047
1071
1082
910
922
930
942
947
954
961
986
993
1010
1019
1036
1055
1084

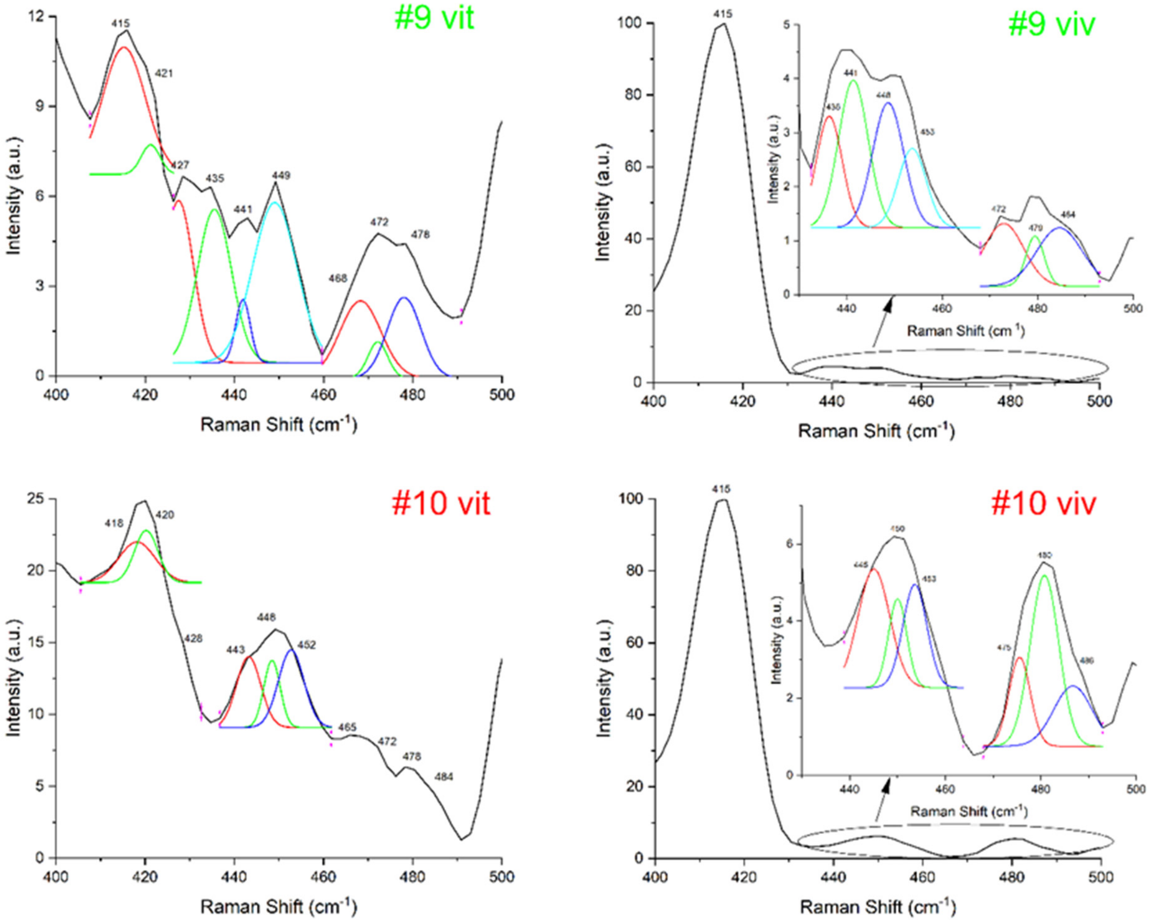

#9 vit
#9 viv
#10 vit
#10 viv
Intensity (a.u.)
Raman Shift (cm-1)
415
421
427
435
441
449
468
472
478
418
420
428
443
448
452
465
484

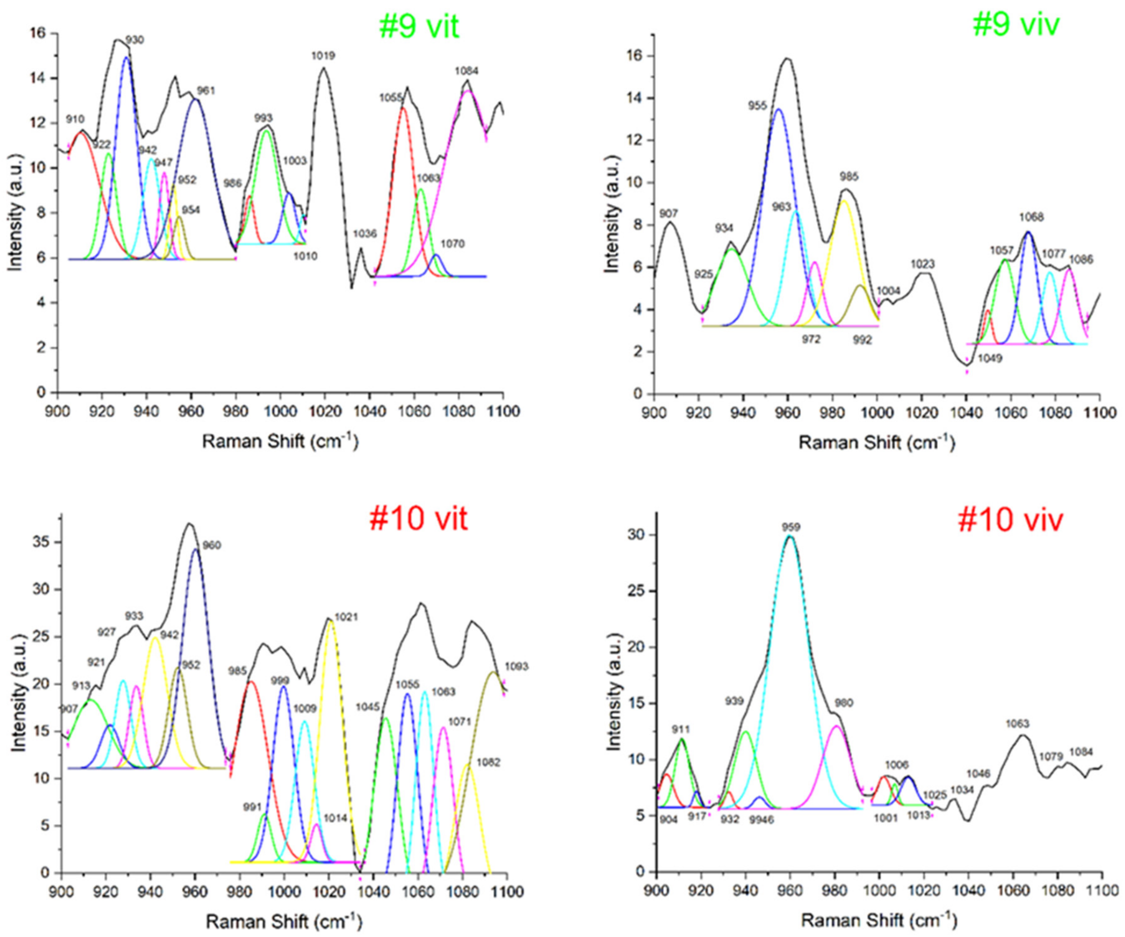

Figure S1. Raman investigation for the patients 3–10, both in vitro and in vivo, analysed in two windows: 400–500 cm^{-1} and 900–1100 cm^{-1}. The numbering stands for: # patient no. in vivo/in vitro.
